# Conjunctival and Lingual mucosal neuromas without multiple endocrine neoplasia type 2B

**DOI:** 10.1016/j.ajoc.2023.101828

**Published:** 2023-03-08

**Authors:** Talia N. Shoshany, Christopher J. Rapuano, Tatyana Milman

**Affiliations:** a Ophthalmology Department, Wills Eye Hospital and Thomas Jefferson University, Philadelphia, PA, USA; b Pathology Department, Wills Eye Hospital and Thomas Jefferson University, Philadelphia, PA, USA

**Keywords:** Conjunctival neuroma, Enlarged corneal nerves, Enlarged corneal nerves MEN2B, Pure mucosal neuroma syndrome, Conjunctival neuroma MEN2B, Pure mucosal neuroma syndrome eye, MEN2B eye, MEN2B conjunctiva, MEN2B cornea, Multiple endocrine neoplasia 2B

## Abstract

**Purpose:**

To report a patient with conjunctival and buccal neuromas and enlarged corneal nerves without Multiple Endocrine Neoplasia 2B (MEN2B).

**Observations:**

A 28-year-old female presented with progressively enlarging bilateral limbal conjunctival growths. Slit lamp examination was notable for enlarged corneal nerves and well-circumscribed gelatinous subepithelial limbal nodules. Systemic examination revealed similar lesions on the tongue. Conjunctival biopsy demonstrated a mucosal neuroma. The patient underwent endocrine workup for MEN2B and genetic testing for the *RET*-proto oncogene mutations, all of which were negative.

**Conclusions and Importance:**

The findings in our patient may be compatible with pure mucosal neuroma syndrome. The pattern of conjunctival neuromas and enlarged corneal nerves should raise concern for MEN2B, a hereditary tumor predisposition syndrome with almost 100% incidence of medullary thyroid cancer, unless prophylactic thyroidectomy is performed. Accurate diagnosis and prompt referral for endocrine and genetic testing is critical. Isolated mucosal neuromas without endocrine manifestations of MEN2B can rarely occur in a “pure mucosal neuroma syndrome,” which is a diagnosis of exclusion in a setting of a negative workup.

## 1 Introduction

Multiple endocrine neoplasia 2B (MEN2B, MIM 164761) is a hereditary cancer predisposition syndrome, characterized by medullary thyroid carcinoma, pheochromocytoma, parathyroid hyperplasia, Marfanoid habitus, and mucosal neuromas.^1^ Classic ocular manifestations include conjunctival (mucosal) neuromas and enlarged corneal nerves. Diagnosis of conjunctival neuroma and enlarged corneal nerves warrants further evaluation for MEN2B.^2^^,^^3^ We describe a young female with conjunctival and buccal neuromas and enlarged corneal nerves, whose endocrine and molecular genetic testing were negative for MEN2B. The findings may be compatible with a rare “pure mucosal neuroma syndrome” (MNS).^2^

## 2 Case report

A 28-year-old female was referred for evaluation of pingueculae in both eyes. The patient reported a five-year history of progressively enlarging bilateral conjunctival growths, associated with mild foreign body sensation in the left eye. She denied trauma, prior ocular surgery, allergies, or significant environmental exposure. Past ocular history was notable for soft contact lens use with good hygiene. Past medical history was unremarkable. Family history was negative for cancer.

**Fig. 1:**
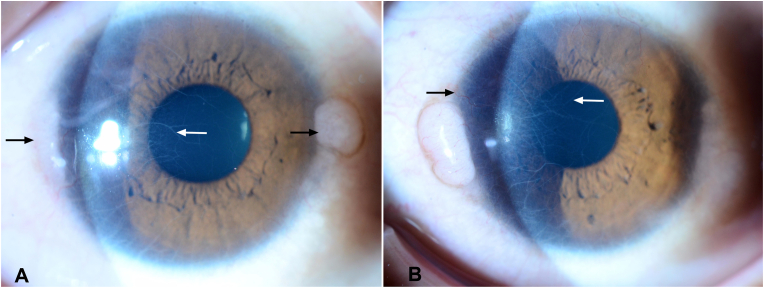
**Conjunctival neuroma, clinical findings. A.** Abruptly elevated gelatinous, non-inflamed nodules at the nasal and temporal limbus of the right eye (black arrows), associated with enlarged corneal nerves (white arrow). **B.** Similar findings in the left eye: Nasal and temporal limbal nodules, prominent corneal nerves (white arrow), and superficial peripheral corneal vascularization (black arrow).

On examination, visual acuity with correction was 20/20 in both eyes. Slit lamp examination was notable for well-circumscribed, white, gelatinous, non-inflamed, subepithelial nodules at the nasal and temporal limbus bilaterally, ranging in size from 1.5 to 3.5 mm, without associated feeder vessels. Peripheral corneal vascularization and prominent corneal nerves were also identified (Fig. 1). The remainder of the eye exam, including evaluation of ocular adnexa, was unremarkable.

**Fig. 2:**
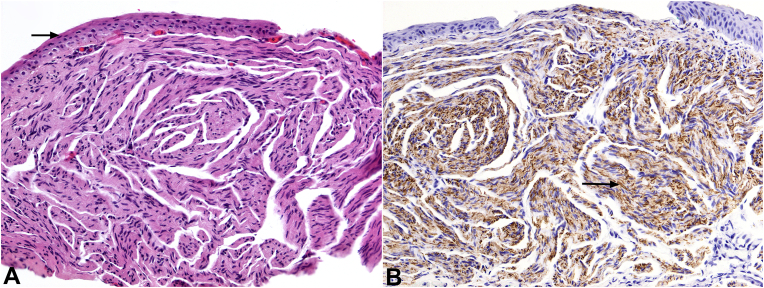
**Conjunctival neuroma, histopathologic findings. A.** A circumscribed proliferation of enlarged, irregular neural bundles beneath the limbal conjunctival epithelium (arrow). **B.** Neurofilament immunohistochemical stain highlights numerous axons (dark brown stain, black arrow), which are approximately equal in number to Schwann cells (blue serpentine nuclei adjacent to axons). [hematoxylin-eosin stain (A), neurofilament stain (B), x100 (A, B)]. (For interpretation of the references to colour in this figure legend, the reader is referred to the Web version of this article.)

**Fig. 3:**
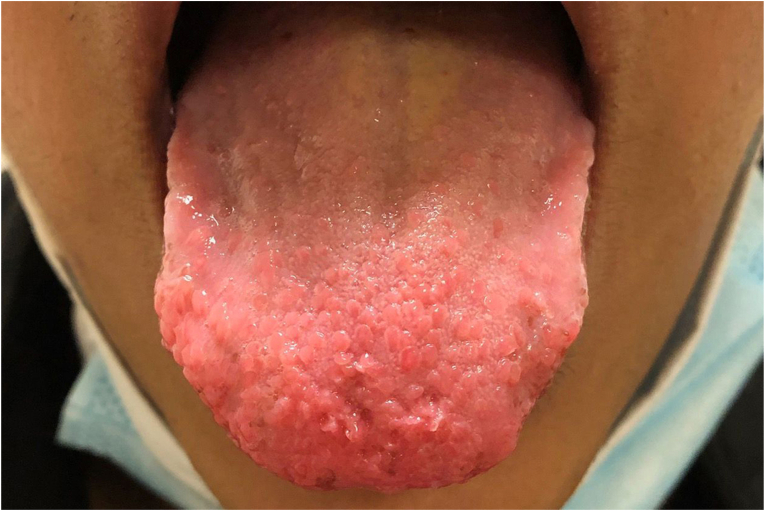
Small nodules on the lateral and dorsal aspect of the tongue suggestive of mucosal neuromas.

Given the atypical appearance of the nodules, decision was made for excision and cryotherapy of the largest and most symptomatic, left nasal limbal lesion. Histopathologic evaluation demonstrated a circumscribed unencapsulated lesion in the subepithelial substantia propria, composed of enlarged neural bundles with equal proportions of neurofilament-positive axons and S100-positive Schwann cells, consistent with a conjunctival neuroma (Fig. 2). Subsequent examination of the patient's oral mucosa revealed similar-appearing gelatinous nodules on the tongue (Fig. 3). Further systemic examination was negative; there was no evidence of Marphanoid habitus or facial dysmorphism.

The combined findings of conjunctival and buccal neuromas and enlarged corneal nerves in our patient were concerning for MEN2B.^1^^,^^2^ The patient was subsequently referred to an internist and a hematologist for further testing, which included thyroid function tests, parathyroid hormone levels, serum calcitonin and carcinoembryonic antigen (CEA), serum and 24-h urine catecholamines, and thyroid gland ultrasound, all of which were within normal limits. Targeted genetic testing did not identify *RET* proto-oncogene mutations. In light of the negative systemic workup for MEN2B, a provisional diagnosis of MNS was rendered. Because the long-term outcomes of MNS are not well-delineated,^2^ the patient is under surveillance for development of endocrine neoplasia. Twelve months following surgery she had no recurrence of the neuroma.

The study and data accumulation were carried out with approval from the Institutional Review Board, Informed Consent for the research was obtained from the patient, and the study is in accordance with HIPAA regulations.

## 3 Discussion

Multiple endocrine neoplasia (MEN) syndromes are autosomal dominant hereditary tumor predisposition syndromes.^1^ MEN2 is caused by gain of function *RET* proto-oncogene mutations on chromosome 10q11.21 and is characterized by medullary thyroid carcinoma and pheochromocytoma, with or without parathyroid hyperplasia (MEN2A vs 2B).^1^ MEN2B also features Marfanoid habitus, musculoskeletal abnormalities, and mucosal (oral, gastrointestinal, and conjunctival) neuromas.^1^ Ocular findings in MEN2B include neuromas in the palpebral and limbal/bulbar conjunctiva, prominent perilimbal vessels, and enlarged corneal nerves.^2^^,^^3^

The differential diagnosis for non-inflamed subepithelial limbal nodules includes degenerations such as pingueculae, deposits such as amyloid, and neoplasms including epibulbar dermoids and neuromas.^4^ The abruptly elevated shape of the nodules is not typical of pingueculae. The acquired nature of the lesions and bilateral nasal and temporal location are not typical of epibulbar dermoids. Histopathologically, conjunctival neuromas are characterized by subepithelial circumscribed proliferation of enlarged neural bundles surrounded by perineureum, with approximately equal proportion of axons and Schwann cells. The diagnosis of a clinically suspected conjunctival neuroma may be supported by anterior segment optical coherence tomography (OCT) and may not require excisional biopsy.^5^ OCT may show subepithelial areas of mixed reflectivity and whirling with unremarkable overlying epithelium – features that are not seen in ocular surface squamous neoplasia, pinguecula, lymphoproliferative lesions, conjunctival nevi, and melanoma.^5^ The differential diagnosis for enlarged corneal nerves includes anatomic variation, dystrophies and degenerations, such as Fuchs corneal endothelial dystrophy and keratoconus, and syndromes including MEN2B.^6^

The ophthalmologist's identification of enlarged corneal nerves in the setting of mucosal neuromas should raise suspicion for MEN2B, warranting a further genetic, endocrinologic, and oncologic workup.^3^ Early diagnosis is important as 100% of patients will develop medullary thyroid carcinoma (MTC) during their lifetime, unless prophylactic thyroidectomy is performed.^1^ MTC arises from the parafollicular cells of the thyroid gland that secrete calcitonin and CEA, which can be used as serum tumor markers.^1^ MTC of MEN2B is particularly aggressive with high incidence of early metastasis.^1^^,^^7^ Pheochromocytomas are benign tumors of the adrenal glands, occurring in 40% of patients with MEN2B.^1^ Pheochromocytomas often arise during the fourth decade and should be resected to prevent consequences of excessive catecholamine release.^1^^,^^8^ Endocrine workup for MEN2B includes thyroid function tests, parathyroid hormone levels, serum calcitonin and CEA, serum and 24-h urine catecholamines, and ultrasound of the thyroid gland.^1^

*RET* proto-oncogene encodes tyrosine kinase receptor with key roles in cell growth, differentiation, and survival. *RET* is widely expressed in neural-crest derived and neuroendocrine tissues, including neurons, thyroid C cells, and the adrenal medulla. Gain of function mutations in *RET* cause constitutive activation of cell proliferative signaling pathways.^1^ Fifty percent of *RET* mutations in MEN2B occur de-novo.^1^ PCR testing for mutations on exons 10–16 of the *RET* proto-oncogene is the gold standard genetic diagnostic test for MEN2.^1^

MNS diagnosis is rendered for those rare patients, who feature mucosal neuromas and enlarged corneal nerves, but lack endocrine manifestations of MEN2B and *RET* mutations.^2^^,^^9^ Neuromas in MNS are primarily limited to non-ocular sites.^2^^,^^9^ To our knowledge, our patient is the third described in the literature, who features both conjunctival neuromas and enlarged corneal nerves.^2^^,^^9^ Most, but not all, patients with MNS have Marfanoid habitus.^2^^,^^3^^,^^9^ *SOS1* frameshift mutations (not evaluated in our patient) have been reported in MNS.^10^ Prophylactic thyroidectomy is not warranted in these patients, although regular monitoring is still advised.^2^^,^^3^ Although *SOS1* mutation status is not known in our patient, presence of mucosal neuromas and enlarged corneal nerves in a setting of negative endocrine workup and *RET* mutation studies is compatible with MNS. Because the long-term outcomes of MNS are not well-delineated due to rarity of this syndrome, our patient will be under oncologic surveillance.

## 4 Conclusion

In conclusion, we describe a patient with MNS, a rare syndrome characterized by mucosal neuromas, including conjunctival neuromas, and enlarged corneal nerves, without endocrine and genetic findings diagnostic of MEN2B. It is essential to recognize that MNS is a diagnosis of exclusion, which can be rendered only after appropriate workup for MEN2B.

## Patient consent

Written consent to publish this report was obtained.

## Funding

This report received no funding.

## Authorship

All authors attest that they meet the current ICMJE criteria for Authorship.

## Declaration of competing interest

The following authors have no conflicts of interest to disclose: Talia Shoshany, Tatyana Milman, Christopher Rapuano.
